# Report of Cases of Appendicitis, with Remarks

**Published:** 1891-12

**Authors:** Joseph Price

**Affiliations:** Physician in charge of the Preston Retreat; Fellow of the American Association of Obstetricians and Gynecologists


### Buffalo Medical and Surgical Journal

Vol. XXXI. DECEMBER, 1891. No. 5.

### Original Communications.

### A DISCUSSION OF APPENDICITIS.[1]

FIRST PAPER.

### A REPORT OF CASES OF APPENDICITIS: WITH REMARKS.

By JOSEPH PRICE, M. D.,

Physician in charge of the Preston Retreat; Fellow of the American Association of Obstetricians and Gynecologists.

The first case I report from memory. It occurred in Ohio eleven months ago.

Case I.—A physician telegraphed me that he had a case of appendicitis of two weeks' standing, and asked me to come at once. I wired him that I would return by way of his city in five days. He permitted the man to remain quietly in bed. I saw him at the end of the twenty-first day, leaking, with a rapid, feeble pulse, and, I think, a subnormal temperature. The patient was twenty-seven years old, and previously had been a vigorous man. The abdomen was greatly distended, and there was marked fluctuation on both sides below the umbilicus. There seemed to be a perfect inflammatory diaphragm at that point. He was placed on the table, a lateral incision made, and two gallons of muddy, pussy fluid escaped. The irrigator, twelve inches long, passed its full length, extending to the left iliac fossa. Two pitchers of water were used, and two large gauze drains, with a glass tube between them, were also used. No stitches were placed. The patient was put in bed, and made a rapid recovery.

Case II.—Mrs. L., aged thirty-two years; married eleven years; no children; no miscarriage; patient of Dr. John T. Hampton. Appendicitis with perforating ulcer of cecum; multiple pus pockets; extensive adhesions to omentum, large and small bowel. Appendix removed. Perforating ulcer of cecum freshened as in vesico-vaginal fistula and closed; stump of appendix inverted; irrigation and drainage, with

---

1. Papers read at a Special Meeting of the Philadelphia County Medical Society, September 28, 1891.

speedy recovery. This patient had suffered recurring attacks, was greatly emaciated, feeble pulse, and slight elevation of temperature. She has gained over thirty pounds since the section.

CASE III.—Mr. H., aged forty-two years. Operation May 5, 1891. A pint of foul pus, fecal in odor ; omentum and adherent loop of ilium ; irrigation ; two tubes used in drainage ; gauze drains. Discharged May 29, 1891.

CASE IV.—Mrs. N., aged twenty-five years. Eleven weeks since confinement. Operation July 4, 1891. Appendicitis of eleven weeks' standing. Appendix removed. Adhesions to cecum, bladder, and uterine walls, all freed. Irrigation and drainage. Patient discharged August 6, 1891.

CASE V.—Mrs. S., aged thirty years ; married eight years. Painful, irregular, and profuse menstrual history ; confined to her bed for the past fifteen months. Appendicitis, with double pyosalpinx. Operation July 6, 1891. Removal of appendages and vermiform appendix ; cyst of left ovary size of orange ; universal adhesions separated ; bowel stitched ; six inches of ileum coiled and firmly adherent to uterus, bladder, and sigmoid. Left tube discharging through ileum ; copious irrigation ; glass and gauze drains. Discharged August 6, 1891.

CASE VI.—Miss W., aged twenty-eight years. Year previous had nephritis and cystitis ; later had severe pain in right and left ovarian regions, paroxysms becoming more severe and frequent ; pain at micturition and stool ; catamenia gradually of shorter duration ; loss of appetite and insomnia. Operation August 20, 1891. Appendicitis and double pyosalpinx. Cyst of left ovary size of hen's egg ; cyst of right ovary size of orange ; adhesions universal. Appendix, distended by concretions and two drachms of fluid, firmly adherent to ileum. Clean removal of appendix and uterine appendages ; irrigation and drainage. Discharged September 19, 1891.

CASE VII.—C. V., aged thirty-two years ; married eight years ; seven children ; Polander by birth ; resident Lucerne county, Pa. Last confinement, January, 1891. Acute mania, no assignable cause: probably puerperal. Indications of abdominal trouble for some time ; went to bed July 23d with pain, tenderness in right iliac region ; tympanitis. Section August 14, 1891. Multiple pus sacs and extensive adhesions to omentum, ileum, bladder, and uterus ; the pus pockets extended from cecum to bladder. Appendix completely disorganized and floating in a pool of pus ; freeing of all adhesions ; irrigation and drainage ; stump of appendix inverted ; stitching of ileum and cecum at disorganized points. She did fairly well for five days; died the sixth day. Postmortem : Intestines in neighborhood of operation quite gangrenous ; a general peritonitis. A large quantity of fetid pus in enlarged right inguinal canal ; also in pocket between bladder and uterus.

*Nomenclature.*—The old terms were arrived at by examining old, neglected cases, often post-mortem. Recently but one term—appendicitis—is used before laparatomy, before post-mortem, because those two performances prove that so far as the gravity, intensity, and extent of the disease are concerned, the symptoms are unreliable and inadequate; further, abdominal sections and post-mortems have determined what the treatment should be—that is, surgical—under a surgeon from its inception; hence the name indicating and impressing the nature of the disease and the character of the treatment—that is, appendicitis surgicalis. Idiopathic peritonitis indicates nothing; it is an empty term.

The terms typhlitis, perityphlitis, paratyphlitis, extraperitoneal abscess of the right iliac fossa, are useless except to indicate a secondary or late process, originating, without exception, in inflammation of the vermiform appendix.

*Symptoms.*—The most constant and valuable signs are:

1. History of sudden onset.

2. The point of greatest sensitiveness to pressure, exactly localized over the base of the appendix.

3. Fever as indicated by the thermometer varies, usually low.

4. Rigidity of right abdominal muscles, constant.

5. Constipation.

6. Edema, overlying a deep abscess, in the right iliac fossa in neglected cases.

7. Shock, more or less profound, usually occurs where perforation happens early and suddenly; it is followed by chill, vomiting and collapse.

There are no special signs of perforation if it takes place late, after adhesions have formed. If perforation occurs late, and the adhesions are imperfect, we find shock.

The symptoms should be studied most carefully at the end of the first twenty-four hours.

8. Pain is misleading; often referred to epigastrium alone; to umbilical region sometimes; it is often slight.

9. Tympanitis is variable; it depends on the state of the bowels; it indicates intestinal paresis—if it comes on rapidly it is unfavorable; it is often the result of opium.

10. Percussion not necessarily dull; there may be a tympanitic note from gas in an overlying intestine.

11. Over-extension of right thigh gives pain.

12. Cough is avoided.

13. Tumor inconstant the first two days.

14. Pulse indicates severity and increase; it shows constitutional disturbance.

15. Chill and vomiting inconstantly accompany the initial pain.

16. A prodromal stage of abdominal discomfort (about a week) is frequent.

17. Flexion of hip-joint not marked except in neglected cases.

The symptoms are not commensurate with the gravity, intensity, or fatality of the disease.

*Diagnosis.*—Early diagnosis is of the greatest importance in reference to treatment and result.

1. Find with tip of finger—using firm pressure—point of sensitiveness (exact point of greatest sensitiveness); it will be in an adult one and a half to two inches inside the right anterior superior spinous process of the ileum, on a line drawn from that process to the umbilicus—in children, according to size, less distant from the spine of the ileum. (McBurney, confirmed by Weir and others.)

2. Rectal examination is of no value early.

3. Difficult to diagnosticate the first twenty-four hours, because few symptoms present.

4. Subjective pain is of little or no value.

5. Constitutional symptoms are far inferior to local signs in forming an accurate diagnosis.

6. Have patient cough.

7. The hypodermic needle should never be used as an aid to diagnosis.

8. Medical men (physicians) no longer diagnosticate these cases —in the start—as simple obstruction of the bowel.

9. Diagnosis by exclusion is the only safe method.

10. The important points—after the disease has been pronounced appendicitis—is to diagnosticate it as an operative or a non-operative case.

*Points in deciding operative or non-operative character of the case:*

1. Do not operate on the first day—usually—because the number of mild cases is undoubtedly large. One operator (McBurney) saw thirteen cases in one year, too mild for operation.

2. If nausea disappears in twelve hours; if tenderness on pressure not increased in twelve hours; if temperature normal, or not above 100° in the mouth; if pulse normal or but little accelerated; if patient moves in bed with ease;—recovery without operation probable.

*Prognosis.*—No positive prognosis can usually be given in the first twelve hours. If we wait for "strong evidence" of perforation, abscess, general peritonitis, rapid, weak pulse, anxious respiration, distension of abdomen, though the operation is made, the patient will not recover.

Surgery has sometimes been successful even where there has been—

(*a*) General suppurative peritonitis;

(*b*) Septic paresis of the intestines;

(*c*) Multiple abscesses in the peritoneal cavity.

Such cases as these, however, usually die, and are classed as hopeless.

Where there is no exhaustion, no general sepsis, no debility from long abstinence from food, no prolonged vomiting, the prognosis is good.

It is a serious, often a difficult, operation.

"Abscess, wherever it is, and however well it may appear to be surrounded by protecting plastic deposits, is a constant menace to life, as evidenced abundantly by its spontaneous opening into the abdominal cavity, the venous canals, the bladder, and the chest cavity, as well as externally and into the intestinal canal."—*Prof. Bridge.*

Pus will form whether there be perforations or none; 145 autopsies had pus (Matterstock); 125 autopsies showed pus (Fenwick, quoted by Keen); 100 autopsies had pus (Weir, quoted by Keen).

Sepsis is hopeless for medicine, and nearly hopeless for surgery.

Sepsis begins before the end of the third day as a rule.

Death from sepsis on the third, fourth, and fifth day is the rule.

The prognosis as to day of death, without operation, in perforative cases is: one-third die between the second and fifth day.

Proof of 176 cases: Eight cases died on the second day; twenty-eight cases died in first three days; forty-six cases died in first four days; sixty cases died in first five days. (Fitz.)

Experience shows no danger existing of infecting the healthy peritoneum in the course of an operation on the appendix.

*Essentials of operative success external to the patient.*—For success the operation requires certain conditions external to the patient, namely: 1. General surgical skill. 2. Good assistants.

*Time of operation.*—"No cases in surgery, saving, perhaps, hemorrhage from large wounded vessels, require more prompt interference" (surgical).—*Keen.*

But one out of twenty-three early laparatomies died. (McBurney.)

"By waiting till the seventh, eighth, or ninth day to operate, a majority only, all more or less dilapidated, will have passed many dangers; not a few will be waiting the knife, that they may start on the road to recovery."

Operate early (before sepsis begins), says one of wide experience, so the operation may not be an autopsy.

Not, however, on the first day, generally speaking. At the end of thirty-six hours, if a tumor has formed, or if symptoms indicate increase in severity or extent of the disease. Before thirty-six hours, if sudden perforation.

If collapse or shock should be very profound, there may be need to stimulate and wait for reaction before operating.

Statistics of time of early operations: Twenty-four cases (early laparatomies), six done on second day; fourteen done on third day; two done on fourth day; two done at the end of one week. Result, twenty-three recoveries, one death. (McBurney.)

*Definition of early operation.*—"By early laparatomy is meant operation before the pathological process has reached an advanced stage. It is not measured by time; it is before (1) general septic peritonitis has begun; (2) before pus flowed freely into the pelvis; (3) before complete septic paresis of intestines has set in."

An early operation is done at a time when the removal of an actually diseased appendix is capable of putting an end at once to an active disease, which has already become clearly defined, and which threatens life.

*Origin of early operation.*—"The early stage operation, or the operation for recurrence, if not of exclusively American origin, has been developed and established by American experience," is true.

*Kinds of incisions.*—A choice is to be made as to the kind of incision—that is, between, first, a free opening into the general peritoneal cavity, and, second, an opening limited to evacuation of the contents of an abscess without exposure of the peritoneum.

*When to use the different kinds of incisions.*—The free opening into the general peritoneal cavity is used (1) in quiescent state after recurrent attacks; (2) in early stage of progressive attacks, with or without abscess or general peritonitis; (3) in later stage, when abscess has formed in one of the rarer positions, and is not adherent to anterior abdominal wall.

The "limited opening" is done in the later stage of the affection for simple evacuation.

*Sites for incision.*—There is a choice of site for free incision as follows: (1) median line below umbilicus (Gerster); (2) outer margin of right rectus muscle below level of umbilicus (Sands).

There are several places at which the "limited incision" may be made; they are (1) parallel to Poupart's ligament, about one inch above it, to above and beyond anterior spine of the ileum (Willard Parker's oblique incision); (2) through floor of iliac fossa; (3) through rectum.

*When the different sites are to be chosen.*—The median line is chosen as a site for the incision in cases of doubtful diagnosis for special indications. The lateral incision is generally chosen for early operations. The oblique incision is made for well-developed abscesses, mainly adherent to the anterior abdominal wall (Réclus).

*Advantages of the different incisions.*—The lateral incision is preferred, because (1) it lies directly over the root of the appendix; (2) it exposes the field of operation more favorably than the median; (3) it creates a shorter, a less exposed, line of drainage.

The advantages of the median incision are, (1) greater probability of not encountering adhesions between the anterior wall and the intestines, in the line of incision; (2) easier access to all parts of the peritoneal cavity for washing and for drainage.

*Instruments.*—Of the instruments to be used, there are but those needed in other major operations.

*Adhesions, limiting.*—Adhesions make appendicitis a local, circumscribed disease; make it an intra-peritoneal disease generally, *i. e.*, nearly always. When they are absent, a diffused mischief is the consequence.

*Adhesions prevent resolution.*—They are left by an attack that apparently ends in recovery; render resolution an absurd term.

*Adhesions cripple organs or parts.*—They may bind appendicitis to (1) parietal peritoneum; (2) omentum; (3) intestines; (4) vessels; (5) itself; (6) in short, to any and all surrounding structures.

*Adhesions cause new trouble.*—When slightly provoked, they set up new trouble.

*Adhesions cause strangulation.*—They lead to strangulation of omentum, bowel, etc.

*Adhesions, frequency of.*—Thirty-five per cent. of all post-mortem examinations made show them. (Toft).

Of 26 cases 6 cases had no adhesions, 6 cases had slight adhesions, 6 cases had marked adhesions—that is to say, 76 12-13 per cent. of the 26 cases had slight, medium, and marked adhesions. (Weir.)

*Adhesions, treatment of.*—Free adhesions by finger.

*Treatment, by whom.*—The physician shares responsibility with the surgeon as early as possible. From the inception of the disease, through the operation to the end, the surgeon and physician should not be separated ; they should see the case together.

*Treatment, washing.*—It has been asserted that hot water is (1) not stimulating ; (2) not cleansing in irrigation ; (3) its use soon followed by depression, marked ; (4) its contact with the peritoneum injurious ; (5) that sponging is more quickly done than irrigation ; that, it being impossible to cleanse hands with soap, water, brush, "rinsing (irrigation) is ridiculous." The above statements are false.

*Treatment where pus is found.*—If pus is found in open pockets, wash out abscess cavities, seek appendix, tie, cut off, invert, cover with or by Lembert's sutures through the outer layer of cecum, pack space between incision and abscess with gauze and iodoform, drain by rubber tube or rubber and glass ; partly close wound, withdraw packing from between intestines on first and second day; withdraw tube and remainder of packing when circumstances indicate.

*Treatment, counter-drain.*—Counter-opening for drainage in flank, above crest of ileum, or through rectum, or where a sinus.

*If little trouble found.*—The removal of a but slightly diseased appendix cuts off the possibility of a recurrence, and is a good thing.

*Where nothing found, i. e., an exploratory operation.*—"Exploratory operations will result in fewer deaths by far than the expectant delay which has heretofore been the general rule." (Keen.)

Exploratory operation " carries with it less danger than the disease "; it has but few risks.

*Treatment of stump.*—To sear mucosa of stump with pure carbolic acid or Paquelin's cautery is lowering an already devitalized part.

To ligate appendix : Invaginate stump, and suture after Lembert, is a good way.

*Site of origin.*—The disease originates in the appendix. (McBurney and McMurtry.)

*Seat of Perforation.*—The point of perforation is usually at the free end of the appendix ; sometimes the whole part is amputated.

*Perforation, frequency of.*—Of 146 cases (Matterstock), 132 cases (60 per cent.) had perforations ; of 129 cases (Fernwick), 113 cases (86 per cent.) had perforations ; of 100 cases (Weir), 34 had perforation ; 200 per cent. of Kümmel's cases had perforations.

*Cause of appendicitis.*—The causes of appendicitis are: 1. Irritating substance, as foods, concretions, foreign bodies. 2. Congenital stricture, occasionally. 3. Strictures at the proximal end, due to ulcerations.

*Concretions, fecal, frequency of.*—Of 146 cases (Matterstock), 63 had fecal concretions ; of 8 cases (Stimson), 1 had fecal concretion.

*Size of foreign bodies that may enter Gerlach's valve.*—Foreign bodies must be small to pass Gerlach's valve and enter the appendix, thus: a cherry-pip may pass with difficulty ; a plum-stone may not enter. And autopsies teach surgery.

*Multiple pus cavities.*—That there are frequently multiple pus cavities, proof: one case (Mikulicz) had 6 pus pockets, opened by three incisions ; another of his cases had 3 pockets, which were opened by incisions.

*Ileo-cecal abscesses.*—They also show ileo-cecal abscess. Proof: of 106 cases (Krafft), 84 had autopsies, 84 had ileo-cecal abscesses.

*Points at which cysts or abscesses of appendix may empty.*—They prove that the abscesses or cysts do and may empty (*a*) into the abdominal cavity ; (*b*) into cecum through Gerlach's valve ; (*c*) into rectum ; (*d*) through Pettit's triangle ; (*e*) above Poupart's ligament ; (*f*) into ischio-rectal fossa ; (*g*) into a coil of intestine; (*h*) through crural ring ; (*i*) into a venous canal ; (*j*) into esophagus ; (*k*) into lung.

*Progress of appendicitis.*—They (laparatomies and autopsies) show three distinct panoramas of progress in the disease.

*Picture 1 of progress.*—(*a*) Irritating substance, catarrh of cecum, or appendix, or both, dilatation of appendix primarily or following dilatation of cecum, contents of bowel enter appendix, fluids are absorbed, solids are left, concretions are formed, and act as mechanical causes for inflammation, and ulceration, upon which follows either amelioration, or relapse, perforation, sepsis more or less extensive, operation or death.

*Picture 2 of progress.*—(*b*) Irritation, catarrh of cecum or appendix, or both, gangrene, obliteration or ulceration, stricture of proximal end during healing of ulcers, distal end patulous, retention sac or cyst forms, which inflames, which empties into cecum, rectum backward, peritoneal cavity, etc.; peritonitis arises, adhesive or local, or universal; quiescent; recurrence; connective tissue becomes involved, omentum, intestines, etc., fixed by adhesions and possible strangulation of omentum, intestines; knife, ligature, irrigation, and drain, must be used or death occurs.

*Picture 3 of progress.*—(*c*) Foreign body; pressure, atrophy, necrosis of epithelium, ulceration, perforation, etc.; knife and its associates, or death.

*Kinds or classes of appendicitis.*—The post-mortem, and the section, show variations in the late or secondary process of appendical inflammation, which have been classified variously.

Professor With subdivides the disease as follows: 1. Peritonitis appendicitis adhesivus. 2. Peritonitis appendicitis localis. 3. Peritonitis universalis. His classification has been largely endorsed, and is recognized under slightly changed names.

Bull distinguishes between (1) catarrhal perityphlitis and (2) suppurative perityphlitis.

Mikulicz classifies it as (1) diffuse septic peritonitis; (2) progressive fibro-purulent peritonitis.

Keen makes five classes, according to progress: 1. Mild form, without perforation, usually no abscesses. 2. Perforative, (*a*) severe, early, fulminant; (*b*) mild, early, suddenly bursts, etc. 3. Perforative, with comfortable abscess, rupturing into hollow viscus, or operated upon. 4. Abscess forming slowly, chronic, weeks, months, years. 5. Recurring.

The sum of all is (McBurney) operative, and non-operative, appendicitis.

*Previous attacks.*—Of 106 cases (Krafft), 24 had previous attacks in 1 to 20 years; 23 per cent. had previous attacks.

*Number of attacks.*—One case (McBurney) had 12 attacks in one year. One case (Treves) had 14 attacks.

*Frequency of the disease.*—35 per cent. of all post-mortems show residua of appendicitis; 36 per cent. (over one-third) of 300 autopsies done at random, revealed diseased appendix (Toft). One case of perityphlitis to 100 of appendicitis (McBurney). Assume that one-third or more of all adults have one or more attacks (Keen).

*Sex most frequently attacked.*—Of 14 cases (Mynter), 1 was a woman; of 24 cases (McBurney), 21 were males, 3 females.

*Age at which it most frequently occurs.*—Of 72 cases (Matterstock), 2 were under two years; 10 were between two and five years; 25 were between five and ten years; 35 were between ten and fifteen years. Of 228 cases (Fitz), 173 cases were below thirty-one years; 207 cases were under forty-one years. Of 24 cases (McBurney), 24 were under thirty-six years. Of 14 cases (Mynter), 1 was under twelve years. Hence it is a disease of early life.

*Results.*—The operation can be done by different individuals under varying conditions, with something like uniform success; proof, the work of Sands, Stimson, Weir, Bull, Senn, Morton, Treves, Hartley, Mynter, Dalton, McBurney.

*Medical and conservative mortality.*—Mortality under conservative treatment is large,—larger than statistics can prove,—for in many fatal cases the origin is unsuspected. Proof: Fitz puts it at 26 per cent.; Stimson puts it at 25 per cent. Of 72 cases, 74 per cent. recovered (Fitz). Of 72 cases about 36 were treated medically, and 11 per cent. died, and 14 per cent. spontaneously evacuated the pus late (Fitz). In medical cases the mortality is now 44 per cent.

*Mortality from early laparatomy.*—Of 24 cases of early laparatomy (McBurney), 23 recovered, 1 died. Of 35 cases of early operation (Weir), 34 recovered, 1 died.

*Mortality from delay.*—For one operation done by mistake ten deaths can be shown from waiting for signs of tumor or peritonitis.

*Acute mischief after quiescence.*—Of 30 cases of acute perforative appendicitis, where recurrence was noticed, 22 cases exploded into abscesses or general peritonitis before the third attack; one case so exploded after the fifth attack.

*Value of medical treatment.*—Medical treatment—*i. e.*, rest and intelligent nursing—is of twofold value: First, in limiting the extent; second, in shortening the duration of mild attacks.

*Items of interest.*—Avoid deep epigastric artery and vein in making lateral incision.

*A point in identification.*—The anterior bundle of unstriped muscles on the colon, which terminates at the base of the cecum, is in sight; it is a good guide to the appendix.

*Abnormalities.*—Anatomical relations of the appendix are sometimes abnormal; it was once found to the left of the median line, above the umbilicus, with a short mesocolon.

*What is recovery without operation?*—Recovery without operation means relief from recurrent pain, and thereafter an improved condition of health for a longer or shorter time (Weir).

*Definition of obliteration of appendix.*—Obliteration of the appendix is narrowing of the appendix, which often follows catarrh of the part.

*Percentage of obliteration of appendix.*—Of 26 cases, 10 gave obliteration.

*Why arrays of statistics?*—The object of bringing statistics, percentages of recoveries, etc., before physicians, is that they may realize the importance of prompt operation (Vander Veer).

*Where Pettit's triangle.*—Pettit's triangle is between the latissimus dorsi and the external oblique muscle.

*Period of activity in literature of appendicitis.*—Much has been written on appendicitis in the last twelve years (1879 to 1891).

*Period of active wrok in appendicitis.*—Much work in appendicitic trouble has been done in the last two years (1889 to 1891).

*Literature.*—BUFFALO MEDICAL JOURNAL, 1879; Transactions of American Surgical Association; Volkmann's Klin. Vorträge, January, 1889; Annals of Surgery, October, 1889; New York Medical Journal, October 25, 1890; Transactions of Medical Society of the State of New York, 1891; Transactions of American Physicians, 1886; Medical and Surgical Reporter, February, 1886; Medical News, March 1, 1890; Lancet, 1889.

The above articles have been read and others in preparation for this paper.

Some of those to whom this article is indebted, and who have largely contributed to the biography of appendicitis, are the following: Drs. Abbe, Buck, Bull, Dalton, Ebebohls, Fenwick, Fowler, Fitz, Gerster, Hartley, Hektoen, Holgh, Hadra, Holmes, Krafft, W. W. Keen, Lange, Morton, McBurney, McMurtry, Mikulicz, Mynter, Matterstock, Mackenzie, Murray, Monks, Porter, Parker, Regnier, Réclus, Roux, Senn, Stimson, Lewis Smith, Sands, Greig Smith, Teale, Tait, Toft, Treves, Vander Veer, With, Weir, and Wyeth.

